# Confirmed Case of Longstanding Respiratory *Francisella tularensis holarctica* Infection: Nebraska, 2022

**DOI:** 10.1093/cid/ciad669

**Published:** 2024-01-31

**Authors:** Rachael Birn, Jeff Hamik, Lana Dayne, Justin Frederick, Amanda Bartling, Peter C Iwen, Adam Wells, Matthew Donahue

**Affiliations:** Epidemiology Unit, Nebraska Department of Health and Human Services, Lincoln, Nebraska, USA; Applied Epidemiology Fellow, Council of State and Territorial Epidemiologists, Atlanta, Georgia, USA; Water, Climate and Health Program, College of Public Health, University of Nebraska Medical Center, Omaha, Nebraska, USA; Epidemiology Unit, Nebraska Department of Health and Human Services, Lincoln, Nebraska, USA; Department of Educational Psychology, University of Nebraska, Lincoln, Nebraska, USA; Communicable Disease Epidemiology Section, Douglas County Health Department, Omaha, Nebraska, USA; Communicable Disease Epidemiology Section, Douglas County Health Department, Omaha, Nebraska, USA; Department of Pathology and Microbiology, Nebraska Public Health Laboratory, University of Nebraska Medical Center, Omaha, Nebraska, USA; Department of Pathology and Microbiology, Nebraska Public Health Laboratory, University of Nebraska Medical Center, Omaha, Nebraska, USA; Pulmonary and Critical Care Medicine Section, Nebraska Methodist Hospital, Omaha, Nebraska, USA; Respiratory Care Program, Nebraska Methodist College, Omaha, Nebraska, USA; Epidemiology Unit, Nebraska Department of Health and Human Services, Lincoln, Nebraska, USA

**Keywords:** *Francisella tularensis holarctica*, chronic tularemia, respiratory

## Abstract

A male patient with distant history of extensive rabbit contact and pulmonary nodules for 6 years developed empyema. *Francisella tularensis holarctica* was isolated from thoracentesis fluid. Retrospective immunohistochemical examination of a pulmonary nodule, biopsied 3 years prior, was immunoreactive for *F. tularensis*. These findings suggest the potential for chronic tularemia.

## Teaser

A male patient with nodules for six years developed empyema. *Francisella tularensis holarctica* was isolated from thoracentesis fluid. Retrospective immunohistochemical examination of a pulmonary nodule, biopsied three years prior, was immunoreactive for *F. tularensis*, suggesting the potential for chronic tularemia.

*Francisella tularensis* was first recognized to cause tularemia in humans in the early twentieth century [1, 2]. Between 2011 and 2019, a 60% increase in disease incidence was observed with nearly 2000 US tularemia cases identified [3]. *F. tularensis* is a gram-negative, pleomorphic, non-spore forming bacterium with 3 subspecies (ssp.): *tularensis, holarctica,* and *mediasiatica*. The ssp. *tularensis* (Type A) is exclusively found in the United States where it accounts for approximately 70% of human tularemia infections, which are most frequently associated with exposure to rabbits and ticks. *F. tularensis* ssp. *holarctica* (Type B) is found throughout the Northern Hemisphere and seldom in Southern Australia, and infection is more frequently associated with exposure to semiaquatic animals (eg, beavers, muskrats, and water voles), environmental exposures to fresh bodies of water and agriculture, and hunting [1, 2, 4, 5]. The ssp. *mediasiatica* has been associated with rodents in Central Asia [1, 2]. After an incubation period of 3–5 days, tularemia typically causes non-specific acute symptoms such as fever, chills, headache, and malaise followed by symptoms associated with exposure route (eg, pneumonia after inhalation of bacteria).

Local health departments investigate cases of tularemia in Nebraska with the assistance of the Nebraska Department of Health and Human Services (DHHS). In Nebraska, an average of 10.2 cases were identified each year over the last 5 years from 2018 to 2022 (n = 51 confirmed and probable cases). Most cases (n = 20, 39%) have been associated with aerosolizing activities (eg, brush-cutting, lawn mowing, high pressure spraying, etc.), tick bites (n = 15, 29%), and hay or harvesting crops (n = 10, 20%).

The Douglas County Health Department and DHHS conducted a detailed epidemiologic investigation after a culture positive for *F. tularensis* was reported in February 2022.

## CASE REPORT

A 67-year-old male, non-smoker with past medical history of controlled hypertension presented with pneumonia in October 2016, characterized by a chest computed tomography (CT) scan with contrast, which revealed bibasilar consolidations, bilateral nodules, and bilateral hilar lymphadenopathy. He was diagnosed with community-acquired pneumonia and treated with 500 mg oral cefuroxime twice daily for 14 days, which resulted in resolution of all symptoms. A follow-up chest CT scan without contrast in March 2017 showed resolution of consolidation and lymphadenopathy but persistence of multiple bilateral pulmonary nodules.

Patient family history was significant for interstitial lung disease in his father (unspecified progressive disease); there was no family history of malignancy. He was a retired construction worker (predominantly the construction of gas stations) and spent significant time outdoors, which included mowing lawns a few days per week in a suburban setting, in addition to time spent watching local outdoor sports. He was a lifetime Eastern Nebraska resident with travel primarily in the Midwest, including several trips to South Dakota each year where he participates in pheasant hunts. He had minimal international travel history—most recently, Ireland in 2007. He did not recall any recent history of tick bites or other infected or swollen arthropod bites, wounds, skin lesions, or contact with small pet bedding, wild animals, rodents, or cats. The patient endorsed substantial exposure to rabbits during his childhood; from 1967 to 1969, the patient and his friends hunted and skinned an estimated >1000 rabbits to sell rabbit meat to his father's coworkers at a local meat processing facility. He did not have rabbit contact since.

**Figure 1.**
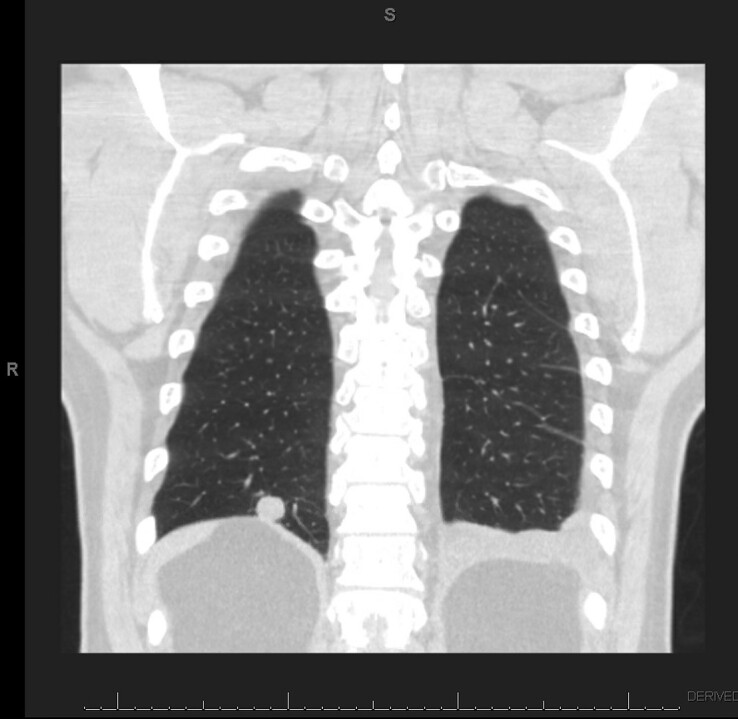
Chest CT obtained in April 2019. Coronal cross-section with right lower lobe nodule and left effusion. Abbreviation: CT, computed tomography.

Surveillance imaging over the next 2 years showed largely unchanged pulmonary nodules until a CT scan in April 2019 prompted consultation with a pulmonologist. The chest CT scan (Figure 1) at that time revealed scattered, non-enlarged mediastinal, hilar, and axillary lymph nodes; enlarging, non-calcified nodules in the right lung; an unchanged, faintly calcified nodule in the left lung; new left basilar opacity; and a new small left pleural effusion. Despite these new findings, the patient was asymptomatic. Given the enlarging non-calcified nodules, a CT-guided biopsy of one of the enlarging nodules in the right lung was performed. Histopathologic examination of the submitted tissue found caseating granulomatous inflammation but no signs of malignancy. Given the absence of malignancy and symptoms, the patient elected continued monitoring with twice yearly scheduled CT scans.

As part of ongoing surveillance in 2021, further evaluation was recommended of the unresolved small left pleural effusion. The patient deferred until February 2022, when a chest X-ray was performed. The left pleural effusion was characterized as persistent and enlarging, prompting a left-sided thoracentesis and culture of the specimen. Pleural fluid studies showed a lymphocyte-predominant exudate with elevated LDH >1000 units/L and a pH of 7.2. After 2 days of incubation, growth was present. Nebraska Public Health Laboratory (NPHL) tested the isolate using a *Francisella tularensis-*specific, real-time polymerase chain reaction (PCR) consisting of 3 target genes. All 3 targets were positive, leading to a presumptive positive result for the isolate. A direct fluorescent antibody (DFA) stain using a polyclonal rabbit anti-*F. tularensis* whole cell serum labeled with fluorescein isothiocyanate (FITC) was used to confirm the isolate as *F. tularensis*. The NPHL used an in-house developed DNA PCR assay to type the organism as ssp. *holarctica* [6]. Cultures for acid-fast bacilli and fungi were negative. Additionally, the complement fixation and the immunodiffusion serological assays were negative for histoplasmosis. The patient was hospitalized and started on 400 mg intravenous gentamicin daily for 7 days and 500 mg oral levofloxacin daily for 14 days. Throughout treatment, the patient remained asymptomatic and without leukocytosis. After consulting with Centers for Disease Control and Prevention (CDC), additional laboratory investigation was pursued, and immunohistochemical evidence of *F. tularensis* was identified from a stored April 2019 paraffin-embedded core needle biopsy of the right lung nodule.

The most recent CT scan in April 2023 revealed the continued absence of mediastinal, hilar, and axillary lymphadenopathy, was negative for pleural effusions and left basilar opacity and did not reveal any new suspicious pulmonary nodules or masses. The patient remained asymptomatic as of April 2023 with most of the previously identified nodular disease showing calcification.

## DISCUSSION

We report a confirmed case of tularemia (Tularemia [*Francisella tularensis*] 2017 Case Definition: https://ndc.services.cdc.gov/case-definitions/tularemia-2017/) in a patient presenting with asymptomatic chronic respiratory infection, as evidenced through immunohistochemistry performed on an April 2019 right lung nodule biopsy tissue and an isolate cultured from a February 2022 left lung empyema. Although abnormal findings were present in pulmonary imaging, the patient experienced no remarkable respiratory symptoms since the pneumonia in October 2016. The association between the 2016 pneumonia and the patient's ultimate diagnosis of tularemia is unclear. The absence of chest imaging prior to 2016 makes the determination of the time and source of original infection difficult. The persistence and evolution of imaging findings since 2016 suggests the patient had chronic tularemia rather than 2 independent acute infections. The resolution of the basilar opacity and empyema, and calcification of the nodules after receiving gentamicin and levofloxacin, further support the diagnosis of chronic tularemia. To our knowledge this is the only known longstanding tularemia infection with laboratory evidence at 2 remote time points years apart.

Descriptions of chronic presentations of tularemia are rare [7–11]. Some acute presentations may become chronic, although asymptomatic or chronic disease caused by Type B could go unidentified and underreported, as these infections are typically milder than Type A infections [2, 4, 9]. Radiographically, respiratory tularemia can present in several ways, including patchy airspace opacities, hilar lymphadenopathy, pleural effusion, cavitation, mediastinal mass, empyema, and others [12]. These might be mistaken for other conditions, including malignancies or pneumonia caused by community-acquired pathogens [13]. Undiagnosed chronic tularemia may have serious outcomes if left untreated. In one instance, an adult male became symptomatic from an undiagnosed chronic tularemia infection with repeated episodes of chemotherapy-induced neutropenia and expired after neutropenic bone marrow transplant [7].

Exposures for the patient could include aerosolization through substantial exposure to infected rabbits during 1967–69, lawn mowing, occupational exposure in construction, hunting, arthropod bite, or from an unknown exposure. Limited 2021 tick testing data in Eastern Nebraska revealed that Type B has been isolated from *Dermacentor variabilis* ticks (unpublished data), which could potentially transmit the pathogen from infected mammals to humans. The disease course timeline is difficult to determine as the patient was either asymptomatic during the [potentially distant] initial infection or was infected in 2016 and recovered from the pneumonia without treatment targeted toward tularemia.

## CONCLUSION

The patient's clinical presentation and evaluation is consistent with longstanding *F. tularensis* ssp. *holarctica* (Type B) infection. Upon epidemiologic investigation, several potential exposure routes were revealed. This case report signals the potential for chronic presentation of type B infections within the United States. The report also highlights the need for a complete medical and exposure history for proper diagnosis, particularly for healthcare providers who may not be familiar with tularemia. An assessment of potential exposures to *F. tularensis* and targeted laboratory testing should be considered in patients with suspicious chronic respiratory findings.

## Notes

***Acknowledgments.*** The authors thank the patient for his contribution to this investigation and follow-up for this report. They thank the Centers for Disease Control and Prevention (CDC) for their assistance with testing and interpretation of the 2019 core needle lung biopsy specimen.

***Supplement sponsorship.*** This article appears as part of the supplement “Tularemia: Update on Treatment and Clinical Findings,” sponsored by the Centers for Disease Control and Prevention.

## References

[1] Sjöstedt A . Tularemia: history, epidemiology, pathogen physiology, and clinical manifestations. Ann N Y Acad Sci 2007; 1105:1–29. 17395726 10.1196/annals.1409.009

[2] Telford SR 3rd , Goethert HK. Ecology of *Francisella tularensis*. Annu Rev Entomol 2020; 65:351–72. 31600457 10.1146/annurev-ento-011019-025134 PMC8300880

[3] Bishop A , Wang H-H, Donaldson TG, et al Tularemia cases increase in the USA from 2011 through 2019. Curr Res Parasitol Vector Borne Dis 2023; 3:100116. 36865594 10.1016/j.crpvbd.2023.100116 PMC9972391

[4] Larson MA , Abdalhamid B, Puniya BL, Helikar T, Kelley DW, Iwen PC. Differences in blood-derived *Francisella tularensis* type B strains from clinical cases of tularemia. Microorganisms 2020; 8:1515. 33019689 10.3390/microorganisms8101515 PMC7600085

[5] Kursban NJ , Foshay L. Tularemia acquired from the pheasant. J Am Med Assoc 1946; 131:1493. 20994375 10.1001/jama.1946.02870350025006

[6] Larson MA , Sayood K, Bartling AM, et al Differentiation of *Francisella tularensis* subspecies and subtypes. J Clin Microbiol 2020; 58:e01495-19. 31941692 10.1128/JCM.01495-19 PMC7098747

[7] Sarria JC , Vidal AM, Kimbrough RC, Figueroa JE. Fatal infection caused by *Francisella tularensis* in a neutropenic bone marrow transplant recipient. Ann Hematol 2003; 82:41–3. 12574964 10.1007/s00277-002-0570-4

[8] Zayet S , Frechet L, Abdallah YB, et al *Francisella tularensis* infection: variable clinical aspects with persistent pulmonary nodules presentation, a case series of human tularemia in Franche-Comté, France. Ticks Tick Borne Dis 2022; 13:101941. 35338968 10.1016/j.ttbdis.2022.101941

[9] Feldman KA , Enscore RE, Lathrop SL, et al An outbreak of primary pneumonic tularemia on Martha's Vineyard. N Engl J Med 2001; 345:1601–6. 11757506 10.1056/NEJMoa011374

[10] Martone WJ , Marshall LW, Kaufmann AF, Hobbs JH, Levy ME. Tularemia pneumonia in Washington, DC. A report of three cases with possible common-source exposures. JAMA 1979; 242:2315–7. 573806 10.1001/jama.242.21.2315

[11] Roth K , Chelikam N, Rathore H, Chittivelu S. An uncommon presentation of pulmonary tularemia: a case report and literature review. Cureus 2022; 14:e30379. 36407204 10.7759/cureus.30379 PMC9667831

[12] Rubin SA . Radiographic spectrum of pleuropulmonary tularemia. AJR Am J Roentgenol 1978; 131:277–81. 98007 10.2214/ajr.131.2.277

[13] Wawszczak M , Banaszczak B, Rastawicki W. Tularaemia—a diagnostic challenge. Ann Agric Environ Med 2022; 29:12–21. 35352900 10.26444/aaem/139242

